# A cross-sectional study on the association between vitamin D levels and caries in the permanent dentition of Korean children

**DOI:** 10.1186/s12903-018-0505-7

**Published:** 2018-03-13

**Authors:** In-Ja Kim, Heung-Soo Lee, Hyun-Jeong Ju, Ja-Young Na, Hyo-Won Oh

**Affiliations:** 1 Department of Preventive and Public Health Dentistry, Wonkwang University, #460 Iksandae-ro, Iksan, Jeonbuk 54538 Republic of Korea; 2 WBMI and Institute of Wonkwang Dental Research, College of Dentistry, Wonkwang University, #460 Iksandae-ro, Iksan, Jeonbuk 54538 Republic of Korea

**Keywords:** Children, Dental caries, Vitamin D

## Abstract

**Background:**

A recent study in Canada reported that vitamin D deficiency is associated with dental caries. Because Koreans have been reported to be deficient in vitamin D, we investigated the relationship between dental caries and reduced serum vitamin D levels in Korean children. The purpose of this study was to analyze the relationships between blood vitamin D [25(OH)D] concentrations and dental caries in the permanent dentition of Korean children.

**Methods:**

Data were collected from the Korea National Health and Nutrition Examination Survey performed in 2008–2013. A total of 1688 children (10–12 years of age) were enrolled. Vitamin D intake was measured through analysis of 25-hydroxy vitamin D [25(OH)D] levels. Caries experience in permanent dentition was assessed using the decay-missing-filled teeth (DMFT) index and decayed-missing-filled (DMF) rate. Statistical analyses included complex samples Chi-square tests, complex samples logistic regression analyses, and Pearson’s correlations.

**Results:**

The group with 25(OH) D levels lower than 50 nmol/L had a higher proportion of children with caries in the permanent dentition and permanent first molar than the group with 25(OH)D levels of 50 nmol/L or more. When external factors, such as sex, were controlled, 25(OH)D levels were not significantly correlated with caries, but were significantly correlated with first molar caries. Children with 25(OH)D levels lower than 50 nmol/L were 1.295 times more likely to have first molar caries than those with 25(OH)D levels of 50 nmol/L or more. Additionally, 25(OH)D levels and DMFT were negatively correlated; however, the degree of correlation was not strong.

**Conclusions:**

The association between 25(OH)D and dental caries is still not clear. However, our findings suggested that vitamin D insufficiency may be a risk factor for dental caries.

## Background

Vitamin D plays an important role in maintaining musculoskeletal and dental health through regulation of the absorption of calcium and phosphorus in the small intestine [1, 2]. Hence, excessive or deficient vitamin D influences oral health. Excessive vitamin D induces overgrowth of cement, causes excessive cellular proliferation with cells invading the marrow space of the jaw bone, thickens the inner part of dentin, and results in pulp stones [3, 4]. In contrast, vitamin D deficiency causes unclear lamina dura in primary and permanent teeth, incomplete calcification of dentin, delay of tooth eruption, and spontaneous periapical abscesses without causative factors, such as dental caries, abrasion, tooth fracture, and tooth trauma [5–7]. Furthermore, vitamin D deficiency may also result in pulp horn extension to the dento-enamel junction, widening of the pulp chamber, taurodontism, and dentin dysplasia [5–7]. In addition, because vitamin D is involved in tooth development, vitamin D deficiency leaves teeth vulnerable to dental caries due to tooth enamel defects [8].

Research on vitamin D supplementation and dental caries began with a study by Mellanby et al. [9], who reported that intake of vitamin D- and vitamin A-fortified cereals in children considerably reduced dental caries and hypoplasia. Furthermore, numerous studies over the past couple of decades have provided evidence for the associations among vitamin D supplementation, tooth development, and caries. Recently, Schroth and colleagues evaluated associations with actual circulating levels of 25(OH)D. Measuring 25(OH)D levels is the gold standard for determining vitamin D status [10]. Most studies on vitamin D and dental caries [10–15] have been conducted in children. Schroth et al. [16] reported the association between vitamin D deficiency and dental caries in a national representative sample of Canadian children. In a study in children ages 3–5 years, Pacey et al. [17] reported that the proportion of children with dental caries was considerably lower in those who consumed nutritional supplements containing vitamin D and calcium than in those who did not. Furthermore, Schroth et al. [10] suggested that children of mothers who had markedly low vitamin D levels during pregnancy had higher incidences of enamel hypoplasia and dental caries than those of mothers who had normal vitamin D levels. Similarly, Tanaka et al. [18] reported that sufficient vitamin D intake during pregnancy was associated with a reduced risk of dental caries in children, based on parental self-reports of caries experience in their children”. Despite multiple studies on vitamin D and dental caries, however, the exact mechanism through which vitamin D influences dental caries has not yet been clarified [13].

A previous study in Korean individuals showed that Koreans are prone to serious health risks due to insufficient 25(OH)D levels and that younger generations in particular exhibit a lack of vitamin D [19]. Therefore, in this study, we aimed to identify the association between 25(OH)D levels and dental caries in Korean children.

## Methods

We analyzed the raw data provided by the Korea National Health and Nutrition Examination Survey (KNHANES), conducted from 2008 to 2013. The participants of KNHANES comprise noninstitutionalized Korean citizens residing in Korea. The sampling plan followed a multistage clustered probability design [20]. This nationally representative cross-sectional survey included approximately 10,000 individuals each year as a survey sample and collected information on socioeconomic status, health-related behaviors, quality of life, healthcare utilization, anthropometric measures, biochemical and clinical profiles for noncommunicable diseases, and dietary intake with three component surveys: health interview, health examination, and nutrition survey [20]. The subjects comprised 1688 children aged 10–12 years who participated in blood tests and oral examinations.

To measure 25(OH)D, KNHANES used 25-hydroxyvitamin D assays, which are commonly used for measuring 25(OH)D [21–24]. In this study, the 25(OH)D levels of children (aged 10–12 years) ranged from 12.90 to 122.80 nmol/L, with a mean level of 47.38 nmol/L. The 25(OH)D status was determined based on classifications established in previous studies [25–27]; the four-category classification divided serum 25(OH)D levels into four groups as follows: less than 25 nmol/L (severe deficiency), between 25 and 50 nmol/L (deficiency), between 50 and 75 nmol/L (insufficiency), and greater than 75 nmol/L (sufficiency), whereas the two-category classification divided serum 25(OH)D levels into two groups as follows: less than 50 nmol/L and 50 nmol/L or more.

In this study, caries was analyzed in two ways: 1) by subdividing into patients who experienced caries in the permanent dentition and those who did not and 2) by subdividing into patients who experienced caries in their first permanent molars and those who did not. We analyzed first molars, which are the first permanent teeth to erupt, because the patients in this study were children in the mixed dentition stage. In this study, dental caries experience referred to an experience of caries on at least one tooth. The first criterion was the presence or absence of dental caries involving the permanent dentition. The second criterion was the total count of the number of decayed, missing, or filled teeth in both the primary and permanent dentition (dmft/DMFT score) [16]. External variables were sex, age, household income, and frequency of tooth brushing.

The association between 25(OH)D levels and dental caries was analyzed using complex samples Chi-square tests. Furthermore, we performed complex samples logistic regression analysis to analyze the association between 25(OH)D levels and dental caries after controlling for external variables. The dose-response relationship between 25(OH)D levels and dental caries experience (number of dental caries and number of first molar caries) was examined through Pearson’s correlations. The level of significance (α) was 0.05, and statistical analyses were conducted using SPSS 22.0 (IBM SPSS Statistics, NY, USA). The Wonkwang Institutional Review Board (WKIRB-201605-SB-027) approved this study. The study followed the guidelines of the World Medical Association Declaration of Helsinki (version 2008).

## Results

Table 1 shows the characteristics of the patients. There were more boys than girls, and the most frequent age was 12 years (11.01 ± 0.24 years, mean ± standard deviation). A majority of the patients had 25(OH)D levels lower than 50 nmol/L (61.7%), and 51.7% of patients had caries experience, whereas 49.2% of the patients had first molar caries experience.

**Table 1:** Characteristics of the subjects

Variables	Classification	N^a^	%^b^
Sex	Female	788	46.6
	Male	900	53.4
	Total	1688	100.0
Age (years)	10	531	31.1
	11	583	33.4
	12	574	35.4
	Total	1688	100.0
Household income	Low	375	24.9
	Middle	882	53.1
	High	414	22.0
	Total	1671	100.0
Frequency of tooth brushing (per day)	≤ 1	211	12.8
	2	969	58.1
	3	426	25.3
	≥ 4	73	3.8
	Total	1679	100.0
Vitamin D^c^ (two classification)	< 50 nmol	1038	61.7
	≥ 50 nmol	650	38.3
	Total	1688	100.0
Vitamin D^c^ (four classification)	< 25 nmol	66	4.4
	≤ 25 to < 50 nmol	972	57.3
	≤ 50 to < 75 nmol	588	35.0
	≥ 75 nmol	62	3.3
	Total	1688	100.0
Caries experience in permanent dentition	No	827	48.3
	Yes	861	51.7
	Total	1688	100.0
Permanent first molar caries experience	No	867	50.8
	Yes	821	49.2
	Total	1688	100.0

The data were analyzed using a complex weighted sample design

^a^ Values are expressed as the unweighted N

^b^ Values are expressed as the weight %

^c^ Values are expressed as 25(OH)D levels

Table 2 shows the results of bivariate analysis of the association between 25(OH)D levels and dental caries experience. The group of children with 25(OH)D levels lower than 50 nmol/L had a higher proportion of dental caries experience than the group of children with 25(OH)D levels of 50 nmol/L or more. In accordance with the four-category classification, groups of children with higher 25(OH)D levels had lower proportions of children with caries experience and first molar caries experience.

**Table 2:** Caries experience according to 25(OH)D levels

Variables	Classification	Caries experience in the permanent dentition			Permanent first molar caries experience		
		No	Yes	*P* ^*^	No	Yes	*P* ^*^
Vitamin D (two classification)	< 50 nmol	476 (45.3)	562 (54.7)	0.012	501 (47.6)	537 (52.4)	0.006
	≥ 50 nmol	351 (53.0)	299 (47.0)		366 (55.8)	284 (44.2)	
	OR^a^	0.735			0.721		
Vitamin D (four classification)	< 25 nmol	27 (40.3)	39 (59.7)	0.048	29 (44.6)	37 (55.4)	0.018
	≤ 25 to < 50 nmol	449 (45.7)	523 (54.3)		472 (47.9)	500 (52.1)	
	≤ 50 to < 75 nmol	318 (52.4)	270 (47.6)		330 (55.0)	258 (45.0)	
	≥ 75 nmol	33 (59.6)	29 (40.4)		36 (64.4)	26 (35.6)	

Units: N (%)

The data were analyzed using a complex weighted sample design

^*^P values were determined from complex samples Chi-square tests

^a^Values are expressed as the adjusted odds ratios

Table 3 shows the results of a multivariate analysis of the association between 25(OH)D levels and dental caries experience. There was no association between 25(OH)D levels and caries experience; however, there was a significant association between 25(OH)D levels and first molar caries experience. When controlling for sex, household income, age, and frequency of tooth brushing, children with 25(OH)D levels lower than 50 nmol/L were 1.295 times more likely to have first molar caries experience than children with 25(OH)D levels of 50 nmol/L or more.

**Table 3:** Relationship between caries experience and general characteristics

Variable		Caries experience		First molar caries experience	
		OR^a^ (95% CI)	*P* value^*^	OR (95% CI)	*P* value^*^
Sex	Male	0.819 (0.644–1.042)	0.104	0.841 (0.666–1.063)	0.148
	Female	1.000 (reference)		1.000 (reference)	
House income	High	1.205 (0.879–1.653)	0.247	1.172 (0.855–1.608)	0.323
	Middle	1.000 (reference)		1.000 (reference)	
	Low	1.314 (0.962–1.795)	0.086	1.250 (0.928–1.685)	0.142
Vitamin D (two classification)	< 50 nmol	1.246 (0.975–1.592)	0.079	1.295 (1.020–1.644)	0.034
	≥ 50 nmol	1.000 (reference)		1.000 (reference)	
Age		1.364 (1.184–1.572)	< 0.001	1.241 (1.084–1.420)	0.002
Frequency of tooth brushing		1.057 (0.886–1.262)	0.537	1.020 (0.855–1.218)	0.824

The data were analyzed using a complex weighted sample design

^*^P values were determined from complex samples logistic regression

^a^Values are expressed as the adjusted odds ratios

Table 4 shows the results of the dose-response relationships between 25(OH)D levels and dental caries experience. The correlations between 25(OH)D levels (original level and four-category classification) and dental caries experience (number of caries and number of first molar caries) were analyzed. The results indicated that all 25(OH)D levels were negatively correlated with dental caries experience; lower 25(OH)D levels were correlated with higher numbers of teeth having caries. However, the degree of correlation was weak.

**Table 4:** Correlation of DMFT and 25(OH)D

	DMFT	First molar DMFT
Vitamin D	−.064^**^	−.053^*^
Vitamin D (four classification)	−.065^**^	−.054^*^

Pearson correlation coefficient

^*^P < 0.05 (two-tailed)

^**^P < 0.01 (two-tailed)

## Discussion

In this study, we analyzed the relationships between blood 25(OH)D concentrations and dental caries in the permanent dentition of Korean children. Our result showed that children with lower than 50 nmol/L 25(OH)D had higher DMF rates than children with a 25(OH)D level of 50 nmol/L or more. Moreover, 25(OH)D levels in the blood were not significantly correlated with caries when external variables, such as sex, were controlled. Thus, our findings provided important insights into the relationships between 25(OH)D levels and caries.

Schroth et al. [16] conducted a study with children ages 6–11 years using data from nationally representative samples of Canadian children. In their study, the proportion of children with dental caries experience among children with 25(OH)D levels lower than 75 nmol/L was 56.4%, while that among children with 25(OH)D levels of 75 nmol/L or more was 43.6%; thus, children with 25(OH)D levels lower than 75 nmol/L had a 1.29-fold higher prevalence of dental caries than did children with 25(OH)D levels of 75 nmol/L or more. Moreover, they also showed that approximately 68.5% of children with 25(OH)D levels lower than 50 nmol/L had dental caries experience, whereas 54.8% of children with 25(OH)D levels of 50 nmol/L or more had dental caries experience, indicating a 1.25-fold greater proportion of children with dental caries experience among children with lower 25(OH)D levels. Our findings were consistent with their findings. Additionally, Schroth et al. [15] analyzed the prevalence of severe early childhood caries (S-ECC) in accordance with 25(OH)D levels in young children and found that 44.8% of young children with 25(OH)D levels of 75 nmol/L or more had S-ECC, whereas 75.0% of those with 25(OH)D levels lower than 35 nmol/L had S-ECC, indicating a 1.7-fold higher prevalence among young children with insufficient vitamin D.

Our multivariate analysis of the association between 25(OH)D levels and dental caries experience showed that children with 25(OH)D levels lower than 50 nmol/L were 1.295 times more likely to have first molar caries experience than those with 25(OH)D levels of 50 nmol/L or more. Similarly, Bener et al. [13] conducted a multivariate Poisson regression analysis to predict caries experience in children ages 7–16 years and found that children with 25(OH)D deficiency (defined using reference points similar to those used in our study) were 1.13 times more likely to have dental caries than those with sufficient 25(OH)D levels. In a 10-year follow-up study, Kühnisch et al. [14] conducted logistic regression analysis and Poisson regression analysis on 1048 children residing in Munich, Germany and found that children with low levels of blood 25(OH)D had many restorations related to caries. Furthermore, Schroth et al. [15] reported that young children with 25(OH)D deficiency were 5.33 times more likely to experience S-ECC compared with those having sufficient levels of vitamin D, consistent with the findings of the present study.

Our correlation analysis of 25(OH)D levels and number of teeth with dental caries indicated that 25(OH)D levels and dental caries experience were negatively correlated; however, the degree of correlation was not strong. Bener et al. [13] reported that children ages 7–16 years with 25(OH)D deficiency had higher decayed teeth, missing teeth, filled teeth, and DMFT than those with sufficient levels of 25(OH)D. Furthermore, they also reported that individuals with a family history of 25(OH)D deficiency had higher decayed teeth, missing teeth, filled teeth, and DMFT than those without a family history of 25(OH)D deficiency. Schroth et al. [10, 16] described the correlation between 25(OH)D levels and caries scores. In both of these studies, significant inverse relationships were reported.

This study was a cross-sectional study; therefore, we could not draw conclusions regarding the causative relationships between 25(OH)D and dental caries. In the future, longitudinal studies should be conducted. Notably, although bacteria, diet, and saliva are important factors in the development of caries, these factors were not investigated in the present study. The interaction of vitamin D with minerals, such as calcium, should also be explored in future studies. However, because the data from this study are based on the national statistical data, we suggest that vitamin D should not be excluded from the list of risk factors for dental caries.

## Conclusions

In conclusion, it was difficult to confirm the association between 25(OH)D levels and dental caries experience. However, our findings suggest that 25(OH)D insufficiency may be associated with dental caries.

## Abbreviations

DMF: Decayed-missing-filled
DMFT: Decayed-missing-filled teeth
S-ECC: Severe early childhood caries

## References

[1] Khazai N, Judd SE, Tangpricha V (2008). Calcium and vitamin D: skeletal and extraskeletal health. Curr Rheumatol Rep.

[2] Garcia MN, Hildebolt CF, Miley DD, Dixon DA, Couture RA, Spearie CLA (2011). One-year effects of vitamin D and calcium supplementation on chronic periodontitis. J Periodontol.

[3] Harris LJ, Innes JR (1931). The mode of action of vitamin D: studies on hypervitaminosis D The influence of the calcium-phosphate intake. Biochem J.

[4] Becks H, Collins DA, Axelrod HE (1946). The effects of a single massive dose of vitamin D2 (D-Stoss therapy) on oral and other tissues of young dogs. Am J Orthod Oral Surg.

[5] Seow WK (1984). X-linked hypophosphataemic vitamin D-resistant rickets. Aust Dent J.

[6] Hillmann G, Geurtsen W (1996). Pathohistology of undecalcified primary teeth in vitamin D-resistant rickets: review and report of two cases. Oral Surg Oral Med Oral Pathol Oral Radiol Endod.

[7] Goodman JR, Gelbier MJ, Bennett JH, Winter GB (1998). Dental problems associated with hypophosphataemic vitamin D resistant rickets. Int J Paediatr Dent.

[8] Cockburn F, Belton NR, Purvis RJ, Giles MM, Brown JK, Turner TL (1980). Maternal vitamin D intake and mineral metabolism in mothers and their newborn infants. Br Med J.

[9] Mellanby M, Pattison CL, Proud JW (1924). The effect of dint on the development and extension of caries in the teeth of children. Br Med J.

[10] Schroth RJ, Lavelle C, Tate R, Bruce S, Billings RJ, Moffatt MEK (2014). Prenatal vitamin D and dental caries in infants. Pediatrics.

[11] Mellanby M, Pattison CL (1928). The action of vitamin D in preventing the spread and promoting the arrest of caries in children. Br Med J.

[12] Mellanby M, Pattison CL (1932). Remarks on the influence of a cereal-free diet rich in vitamin D and calcium on dental caries in children. Br Med J.

[13] Bener A, Al Darwish MS, Hoffmann GF (2013). Vitamin D deficiency and risk of dental caries among young children: a public health problem. Indian J Oral Sci.

[14] Kühnisch J, Thiering E, Kratzsch J, Heinrich-Weltzien R, Hickel R, Heinrich J (2015). Elevated serum 25(OH)-vitamin D levels are negatively correlated with molar-incisor hypomineralization. J Dent Res.

[15] Schroth RJ, Levi JA, Sellers EA, Friel J, Kliewer E, Moffatt ME, Vitamin D (2013). Status of children with severe early childhood caries: a case-control study. BMC Pediatr.

[16] Schroth RJ, Rabbani R, Loewen G, Moffatt ME (2016). Vitamin D and dental caries in children. J Dent Res.

[17] Pacey A, Nancarrow T, Egeland GM (2010). Prevalence and risk factors for parental-reported oral health of Inuit preschoolers: Nunavut Inuit child health survey, 2007-2008. Rural Remote Health.

[18] Tanaka K, Hitsumoto S, Miyake Y, Okubo H, Sasaki S, Miyatake N (2015). Higher vitamin D intake during pregnancy is associated with reduced risk of dental caries in young Japanese children. Ann Epidemiol.

[19] Choi HS, Oh HJ, Choi H, Choi WH, Kim JG, Kim KM (2011). Vitamin D insufficiency in Korea--a greater threat to younger generation: the Korea National Health and nutrition examination survey (KNHANES) 2008. J Clin Endocrinol Metab.

[20] Kweon SH, Kim YN, Jang MJ, Kim YJ, Kim KR, Choi SH, Chun CM, Khang YH, Oh K (2014). Data resource profile: the Korea National Health and nutrition examination survey (KNHANES). Int J Epidemiol.

[21] Stamp TC, Round JM, Rowe DJ, Haddad JG (1972). Plasma levels and therapeutic effect of 25-hydroxycholecalciferol in epileptic patients taking anticonvulsant drugs. Br Med J.

[22] Birge SJ, Haddad JG (1975). 25-hydroxycholecalciferol stimulation of muscle metabolism. J Clin Invest.

[23] Haddad JG, Rojanasathit S (1976). Acute administration of 25-hydroxycholecalciferol in man. J Clin Endocrinol Metab.

[24] Weisman Y (2013). Vitamin D deficiency and insufficiency. Isr Med Assoc J.

[25] Bischoff-Ferrari HA, Giovannucci E, Willett WC, Dietrich T, Dawson-Hughes B (2006). Estimation of optimal serum concentrations of 25-hydroxyvitamin D for multiple health outcomes. Am J Clin Nutr.

[26] Holick MF (2007). Vitamin D deficiency. N Engl J Med.

[27] Ministry of Health and Welfare. Dietary reference intakes for Koreans 2015. Sejong: Ministry of Health and Welfare; 2015.

